# Exploring the Causal Links Between 338 Cerebrospinal Fluid Metabolites and Parkinson's Disease

**DOI:** 10.1002/brb3.70815

**Published:** 2025-09-02

**Authors:** Houwen Zhang, Chunrong Li, Jialin Yu, Yu Liang, You Wu, Fangzheng Cao

**Affiliations:** ^1^ Department of Neurology The Second Affiliated Hospital of Zhejiang Chinese Medical University Hangzhou Zhejiang China; ^2^ The Second Clinical Medical College of Zhejiang Chinese Medical University Hangzhou Zhejiang China; ^3^ Center for Rehabilitation Medicine, Department of Neurology Zhejiang Provincial People's Hospital (Affiliated People's Hospital, Hangzhou Medical College) Hangzhou Zhejiang China

**Keywords:** CSF metabolites, glycerophosphoinositol, Mendelian randomization, oxalate, Parkinson's disease

## Abstract

**Background:**

Parkinson's disease (PD) is a progressive neurodegenerative condition characterized by motor impairments and intricate pathophysiological mechanisms. Although cerebrospinal fluid (CSF) metabolites are viewed as potential biomarkers, the primarily observational design of the majority of existing studies limits the ability to draw causal conclusions.

**Objective:**

The purpose of this research was to explore the causal link between CSF metabolites and PD by employing two‐sample Mendelian randomization (TSMR).

**Methods:**

Data on CSF metabolites were acquired from the two longitudinal cohort studies. Information regarding PD was gathered from the International Parkinson's Disease Genomics Consortium. Instrumental variables (IVs) for 338 CSF metabolites were identified based on notable SNPs. Causal impacts were assessed using Mendelian randomization techniques including inverse variance weighted (IVW) analysis and MR‐Egger regression. The analyses included sensitivity assessments to evaluate issues related to heterogeneity and pleiotropy.

**Results:**

Significant causal associations were found between several CSF metabolites and PD. Higher levels of glycerophosphoinositol, sphingomyelin, and oxalate were linked to increased PD risk, while elevated caffeine levels appeared protective. After FDR correction, glycerophosphoinositol, oxalate, and caffeine remained significant. Sensitivity analyses confirmed robustness with no heterogeneity or pleiotropy.

**Conclusion:**

This analysis of MR reveals multiple CSF metabolites that have causal links to PD. Increased levels of glycerophosphoinositol and oxalate are associated with higher risk, whereas caffeine provide protective benefits. These metabolites may act as potential biomarkers and treatment targets for PD.

## Graphical abstract

This Mendelian randomization study systematically screened 338 cerebrospinal fluid metabolites and identified several with potential causal effects on Parkinson's disease, offering new insights into disease pathogenesis.

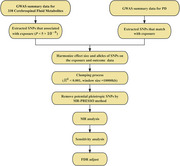

## 1 Introduction

Parkinson's disease (PD) is a neurodegenerative condition that mainly impacts the motor system and progressively deteriorates with time. It is characterized by slowness of movement, stiffness, tremors at rest, and balance issues (Xia and Mao 2012). The global prevalence of PD is increasing, affecting millions worldwide (Tysnes and Storstein 2017). The intricate pathophysiology of PD includes the aggregation of α‐synuclein in the brain along with the degeneration of dopaminergic neurons within the substantia nigra (SN) pars compacta (Dexter and Jenner 2013). Recent research has revealed that metabolites found in cerebrospinal fluid (CSF) could serve as potential biomarkers for neurodegenerative disorders, owing to their direct engagement with the central nervous system and their role in the fundamental pathophysiological mechanisms.

The pathology of PD encompasses several domains, including neuroinflammation (Boyd et al. 2022), mitochondrial dysfunction (Ridler 2018), oxidative stress (Wang et al. 2021), autophagy (Boland et al. 2018), and cellular senescence (Hou et al. 2019). Previous research has highlighted that alterations in various CSF metabolites may influence these pathways, with specific focus on glutathione (Bjørklund et al. 2021; Reyes et al. 2016), lipids (Qiu et al. 2022; Fernández‐Irigoyen et al. 2021), fatty acid β‐oxidation (Li et al. 2024), energy metabolism (Liu et al. 2021; Anandhan et al. 2017), purines (Von Seggern et al. 2020; Morelli et al. 2010), polyamines (Vrijsen et al. 2023; Saiki et al. 2019), tryptophan (Heilman et al. 2020), kynurenine (Venkatesan et al. 2020), and phenylalanine (Molina et al. 1997). However, most of these studies have been observational, making it challenging to establish causality.

Mendelian randomization (MR) employs genetic variations as instruments to assess the causal effects of exposures on outcomes (Bowden and Holmes 2019). This method offers several advantages, including reduced confounding due to the random assignment of genetic variants, minimized reverse causation, and fewer measurement errors, as genetic variants remain stable over time. MR also provides a robust framework for establishing causality, ensuring that the exposure leads to the outcome, rather than the reverse (Emdin et al. 2017).

The objective of the study is to employ two‐sample Mendelian randomization (TSMR) analyses to explore the causal relationships between CSF metabolites and PD, using information gathered from genome‐wide association studies (GWAS).

## 2 Methods

**FIGURE 1:**
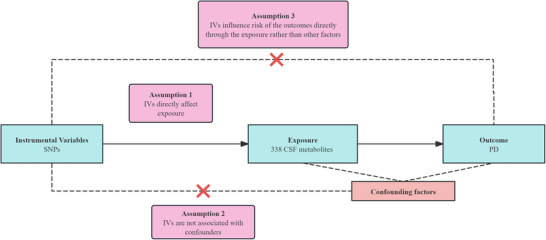
Summary of the study design. CSF, cerebrospinal fluid; MR, Mendelian randomization; PD, Parkinson's disease; SNPs, single nucleotide polymorphisms.

The study design is comprehensively illustrated in Figure 1, highlighting its key components and methodology. This study was reported according to the Strengthening the Reporting of Observational Studies in Epidemiology‐Mendelian Randomization (STROBE‐MR) guidelines.

### 2.1 Data Sources of CSF Metabolites

Data on CSF metabolites primarily originated from two longitudinal cohort studies conducted at the Wisconsin Alzheimer's Disease Research Center (WADRC) (Johnson et al. 2018) and the Wisconsin Registry for Alzheimer's Prevention (WRAP) (Darst et al. 2019). To make the results more applicable, the studies targeted individuals who were cognitively healthy (Panyard et al. 2021). After data cleaning, demographic characteristics across both cohorts were found to be similar. The CSF metabolite and genotype information were imputed and rigorously subjected to quality control measures, resulting in a final dataset that included 291 baseline visits from unrelated individuals of European ancestry, which comprised 338 CSF metabolites (Panyard et al. 2021).

### 2.2 Data Sources of PD

GWAS summary data related to PD were sourced from a large‐scale study by the International Parkinson's Disease Genomics Consortium (Nalls et al. 2019). The study involved a group of 482,730 people of European descent, including 33,674 individuals with PD and 449,056 without. Roughly 17.8 million genetic variants were examined following the implementation of strict quality control and imputation techniques.

### 2.3 Instrumental Variables (IVs) Selection

Identifying single nucleotide polymorphisms (SNPs) with a strong association with the CSF metabolites was key in selecting instrumental variables for this study. A *p* value threshold of less than *p* < 5 × 10^−8^ was used for SNP selection (Zhang et al. 2024). To minimize the influence of linkage disequilibrium, we applied a clumping procedure using the European population reference panel with an *R* ^2^ threshold of < 0.001 and a 10,000 kb window to ensure that selected SNPs were independent (Evans and Davey Smith 2015). In order to mitigate the risk of bias arising from weak instruments, only those SNPs that had an *F*‐statistic exceeding 10 were deemed suitable for inclusion in the final analysis (Storm et al. 2021).

### 2.4 MR Analysis

This research utilized five MR techniques to explore possible causal links between CSF metabolites and PD. These techniques included inverse variance weighted (IVW) (Slob and Burgess 2020), MR‐Egger (Bowden et al. 2015), simple mode, weighted median, and weighted mode (Bowden et al. 2016). The primary method applied for the analysis was IVW. To mitigate the likelihood of Type 1 errors resulting from multiple comparisons, the false discovery rate (FDR) correction was implemented to adjust the significance thresholds for each result set (Storey and Tibshirani 2003).

### 2.5 Sensitivity Analysis

To verify the reliability of the MR estimates, tests for heterogeneity and horizontal pleiotropy were conducted. The evaluation of heterogeneity utilized Cochran's *Q* test (Greco et al. 2015). The investigation of horizontal pleiotropy involved the use of the MR‐Egger intercept test (Burgess and Thompson 2017). A leave‐one‐out analysis was executed to assess the stability of the results (Burgess et al. 2017).

Analyses were performed in R 4.3.1 using the TwoSampleMR, MRPRESSO, and qvalue packages.

## 3 Results

### 3.1 IVs Selection

Table S1 contains the data for the chosen SNPs. A total of 14,167 SNPs were selected as IVs for the 338 CSF metabolites in PD (refer to Table S2). All chosen IVs had *F*‐statistics exceeding 10, with values between 20.65 and 904.75, indicating no weak IVs were present in the analysis.

### 3.2 MR Analyses of CSF Metabolites on PD

**FIGURE 2:**
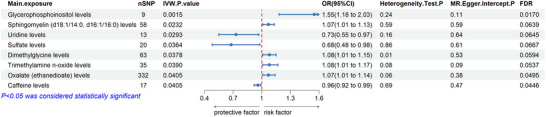
Forest plot depicting the relationship between CSF metabolites and PD risk. The influence of CSF metabolites on the risk of PD is illustrated by the odds ratio (OR) and the corresponding 95% confidence interval (CI). Statistical significance following multiple comparisons was assessed using a false discovery rate (FDR) of less than 0.05. Cochran's *Q* test was employed to evaluate heterogeneity (a *p* value below 0.05 indicates the presence of heterogeneity). The MR‐Egger intercept was utilized to test for horizontal pleiotropy (with *p* < 0.05 suggesting pleiotropy).

**FIGURE 3:**
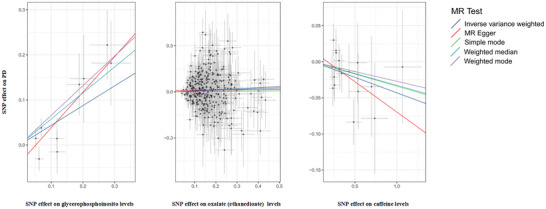
Scatter plot illustrating MR analysis of the correlation between significant CSF metabolites and PD. SNPs that correlate with CSF metabolites are displayed alongside their respective associations with PD (depicted as black dots). Vertical and horizontal lines signify the 95% confidence intervals for the odds ratios of each SNP. The slope of these lines indicates the estimated causal effect, as identified through MR methodologies.

Figures 2 and 3 illustrate the notable links between CSF metabolites and PD, with comprehensive results available in Table S2.

Genetic predispositions to elevated levels of glycerophosphoinositol (OR = 1.55, 95% CI = 1.18–2.03, *p* = 0.0015), sphingomyelin (d18:1/14:0, d16:1/16:0) (OR = 1.07, 95% CI = 1.01–1.13, *p* = 0.0232), dimethylglycine (OR = 1.08, 95% CI = 1.01–1.15, *p* = 0.0378), trimethylamine *N*‐oxide (OR = 1.08, 95% CI = 1.01–1.17, *p* = 0.0390), and oxalate (ethanedioate) (OR = 1.07, 95% CI = 1.01–1.14, *p* = 0.0405) were associated with an increased risk of PD (Figure 2).

In contrast, genetic predispositions to levels of uridine (OR = 0.73, 95% CI = 0.55–0.97, *p* = 0.0293), sulfate (OR = 0.68, 95% CI = 0.48–0.98, *p* = 0.0364), and caffeine (OR = 0.96, 95% CI = 0.92–0.99, *p* = 0.0405) were found to be protective against PD (Figure 2).

After applying the FDR correction, only glycerophosphoinositol, oxalate (ethanedioate), and caffeine remained statistically significant (*P*_FDR_ < 0.05). Other metabolites, such as sphingomyelin, dimethylglycine, trimethylamine *N*‐oxide, uridine, and sulfate showed nominal significance (uncorrected *p* < 0.05) but did not survive multiple testing correction, and should therefore be interpreted with caution (Figure 2).

### 3.3 Sensitivity Analyses

**FIGURE 4:**
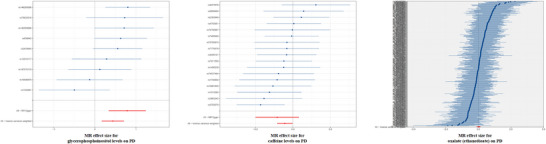
Forest plot representing SNPs in MR analysis of the correlation between significant CSF metabolites and PD. This plot highlights the influence of individual SNPs on PD risk. Each black horizontal line symbolizes the effect estimate for a particular SNP concerning PD risk, computed via the Wald ratio approach. The red line denotes the overall effect estimate for CSF metabolite‐associated SNPs on PD risk, calculated by aggregating the effects of all individual SNPs.

**FIGURE 5:**
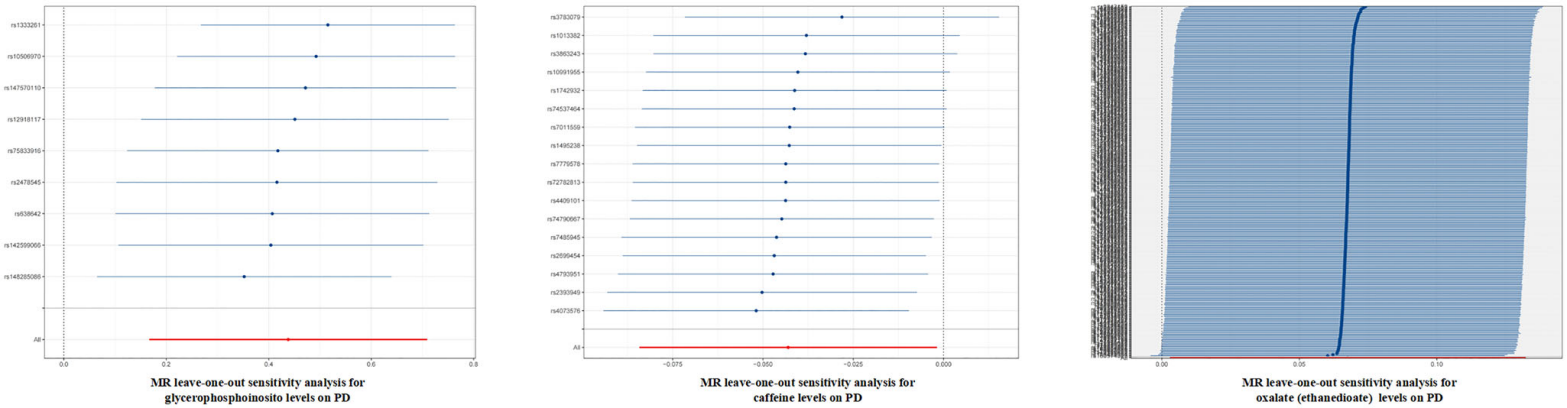
“Leave‐one‐out” Analysis plot for MR analysis of the correlation between significant CSF metabolite levels and PD. In this analysis, each SNP is systematically excluded one at a time, and the meta‐analysis effects of the remaining SNPs are reassessed to observe how excluding each SNP affects the overall results.

Based on Cochran's *Q* test, heterogeneity was not observed among the chosen SNPs for any outcomes that were statistically significant (*p* > 0.05) (Figure 4), as detailed in Table S2. Furthermore, the MR‐Egger intercept test revealed no indications of horizontal pleiotropy or outlier values among the IVs for all CSF metabolites (*p* > 0.05), which is detailed in Table S2. In addition, the leave‐one‐out analysis illustrated in Figure 5 further validated the robustness of our MR findings.

### 3.4 Reverse MR

Reverse MR analysis was performed to assess whether PD might causally influence the levels of the key CSF metabolites. No significant associations were observed for any of the evaluated metabolites, including glycerophosphoinositol (*p* = 0.575), oxalate (*p* = 0.611), caffeine (*p* = 0.779), sphingomyelin (d18:1/14:0, d16:1/16:0) (*p* = 0.779), dimethylglycine (*p* = 0.319), trimethylamine *N*‐oxide (*p* = 0.075), uridine (*p* = 0.175), and sulfate (*p* = 0.980), and others. Detailed results are provided in Table S3.

## 4 Discussion

Our MR analysis identified three CSF metabolites—glycerophosphoinositol, oxalate (ethanedioate), and caffeine—that demonstrated statistically significant associations with PD risk after correction for multiple testing. Specifically, elevated levels of glycerophosphoinositol and oxalate were associated with increased PD risk, whereas higher caffeine concentrations appeared protective. Several other metabolites, including sphingomyelin and dimethylglycine, among others, showed nominal associations (uncorrected *p* < 0.05) but did not remain significant after FDR correction. These findings suggest that alterations in specific metabolic pathways may contribute to PD pathogenesis and highlight a subset of CSF metabolites as potential biomarkers or therapeutic candidates, warranting further validation. Notably, reverse MR analysis did not reveal significant causal effects from PD on these metabolite levels, supporting a unidirectional relationship from metabolite exposure to disease risk.

Glycerophosphoinositols, common metabolites of phosphoinositides, are essential in controlling numerous cellular functions (Corda et al. 2009). Research has previously demonstrated that in PD patients, glycerophosphoinositol levels are increased in the lipid rafts of the frontal cortex (Fabelo et al. 2011), with similar findings observed in fibroblasts with Parkin mutations (Lobasso et al. 2017). Our results align with these findings and provide causal insights through MR analysis. α‐Synuclein has a high affinity for vesicles with acidic phospholipids like glycerophosphoinositols, aiding in the creation of stable multimers (Davidson et al. 1998). These metabolites play a role in controlling vesicle cycling at presynaptic terminals, where there is a high concentration of α‐synuclein (Frere et al. 2012). Elevated glycerophosphoinositol levels may contribute to mitochondrial turnover defects (Lobasso et al. 2017) and enhance NOTCH3 mRNA expression (Luo et al. 2024). Notch‐3 clustering has been connected to neurovascular unit damage, such as pericyte loss and the disconnection of astrocytic end‐feet from brain microvessels, which leads to a more permeable blood–brain barrier (Ghosh et al. 2015). Furthermore, inhibiting Notch signaling has been shown to reduce neurodegenerative changes in PD by decreasing microglial activation and inflammation (Liang et al. 2023). The results together emphasize the significant involvement of glycerophosphoinositol in PD pathogenesis.

Oxalate, a metabolic byproduct of ascorbate (vitamin C) and dopamine in the brain, has been implicated in the pathogenesis of PD (Heller and Coffman 2019). Electron microscopy has revealed the presence of calcium oxalate dihydrate crystals in the substantia nigra of PD patients, suggesting that these deposits may contribute to local inflammation and oxidative stress (Heller and Coffman 2019). In individuals with primary hyperoxaluria, calcium oxalate microparticles have also been observed in the brain and meninges, where they are associated with localized microglial activation (Haqqani 1977). Moreover, in PD mouse models, Raman spectroscopy has identified elevated oxalate accumulation and oxidative modifications in the striatal extracellular matrix, which impaired microglial survival, altered cell morphology, and reduced cytoskeletal tension—indicating that oxalate enrichment may disrupt the neural microenvironment and promote neurodegenerative changes (Freitas et al. 2021). Calcium oxalate crystals are also known to activate the inflammasome (Heller and Coffman 2019), induce oxidative stress via NADPH oxidase activation, and impair mitochondrial function, resulting in cellular injury (Thamilselvan et al. 2009). While no direct experimental evidence currently supports a physical interaction between oxalate and α‐synuclein, the accumulation of oxalate in PD‐relevant brain regions may indirectly contribute to α‐synuclein aggregation and neuronal damage. The documented loss of axons and demyelination in peripheral nerves further underscores the neurotoxic potential of oxalate (Lorenz et al. 2013). The documented loss of axons and demyelination in peripheral nerves underscores the neurotoxic impact of oxalate (Lorenz et al. 2013). Our findings align with this body of evidence, suggesting that oxalate may act as a pathophysiological contributor to PD risk.

For many years, it has been suggested that consuming caffeine might reduce the risk or delay the onset of PD, as plasma levels of caffeine are thought to play a significant role (Ross et al. 2000; Ascherio et al. 2001; Hu et al. 2007). This link has been validated by recent extensive prospective research (Zhao et al. 2024). Our MR results further support the protective role of caffeine, specifically at the CSF level, with robust control for confounding and reverse causation. Mechanistically, caffeine readily crosses the blood–brain barrier and acts as a nonselective antagonist of adenosine A2A receptors, which are highly expressed in the striatum. By blocking these receptors, caffeine modulates glutamatergic neurotransmission, reduces excitotoxicity, attenuates neuroinflammation, and enhances dopamine D2 receptor signaling—effects that contribute to dopaminergic neuron protection and motor function improvement (Chen et al. 2013; Badshah et al. 2019; Reichmann 2023; Song et al. 2024). A2A receptor antagonism has also been shown to modify disease progression in PD animal models (Reichmann 2023). In addition to receptor‐mediated effects, caffeine may directly reduce neurotoxin bioactivity. Molecular docking studies indicate that caffeine forms π–π stacking interactions with neurotoxins such as 1‐methyl‐4‐phenyl‐1,2,3,6‐tetrahydropyridine (MPTP), potentially lowering their bioavailability and neurotoxic potential (Ulanowska et al. 2005). Moreover, estrogen‐related modulation may affect caffeine sensitivity, leading to sex‐specific neuroprotection (Ascherio et al. 2004). Salivary studies have reported that caffeine levels decline with PD progression, independently of levodopa treatment, suggesting that altered caffeine metabolism may reflect disease severity (Leodori et al. 2021). Finally, animal studies show that caffeine administration reduces CSF oxidative stress markers such as malondialdehyde, further supporting its biological activity within the central nervous system (Bandookwala et al. 2019).

Nonetheless, it is important to recognize several limitations. First, even though our MR method enhances causal inference, the relatively limited sample size of the CSF metabolite GWAS could constrain statistical power and the ability to generalize findings. Second, all exposure and outcome summary statistics were derived from individuals of European ancestry, which restricts the generalizability of our findings. It is therefore essential that future studies validate these results in more diverse, multi‐ethnic populations to ensure broader applicability. Lastly, while sensitivity analyses affirm the robustness of our findings, it is not possible to completely rule out residual pleiotropy. Future research that incorporates multi‐omic strategies and mechanistic experiments is essential to clarify the exact pathways through which these metabolites influence the pathogenesis of PD.

## 5 Conclusion

To summarize, our MR analysis revealed possible causal links between CSF metabolites and PD. Higher levels of glycerophosphoinositol and oxalate (ethanedioate) correlated with an increased risk of PD, whereas raised levels of caffeine seemed to offer protective benefits. This study underscores the significance of CSF metabolism in PD and proposes that these metabolites could represent valuable therapeutic targets for future investigative efforts.

## Author Contributions

**Houwen Zhang**: conceptualization, methodology, software, writing – original draft. **Chunrong Li**: methodology, conceptualization, writing – original draft. **Jialin Yu**: methodology, writing – original draft. **Yu Liang**: methodology, writing – original draft. **You Wu**: methodology, funding acquisition, writing – original draft. **Fangzheng Cao**: writing – review and editing, methodology, software, supervision.

## Ethics Statement

This research made use of data that is publicly accessible. Every element of the GWAS received approval from the appropriate Institutional Review Boards, and informed consent was collected from all participants or their designated proxies.

## Consent

The authors have nothing to report.

## Conflicts of Interest

The authors declare no conflicts of interest.

## Peer Review

The peer review history for this article is available at https://publons.com/publon/10.1002/brb3.70815.

## Supporting information




**Supplementary Materials**: brb370815‐sup‐0001‐TableS1.xlsx

**Supplementary Materials**: brb370815‐sup‐0002‐TableS2.xlsx

**Supplementary Materials**: brb370815‐sup‐0003‐TableS3.xlsx

## Data Availability

The GWAS Catalog hosted by the European Bioinformatics Institute supplied summary statistics for CSF metabolites, identified by accession numbers ranging from GCST90025999 to GCST90026336. Data related to PD were sourced from the MRC‐IEU OpenGWAS database (https://gwas.mrcieu.ac.uk). For additional details, please consult the original GWAS studies referenced in our paper.

## References

[brb370815-bib-0001] Anandhan, A. , M. S. Jacome , S. Lei , et al. 2017. “Metabolic Dysfunction in Parkinson's Disease: Bioenergetics, Redox Homeostasis and Central Carbon Metabolism.” Brain Research Bulletin 133: 12–30. 28341600 10.1016/j.brainresbull.2017.03.009 PMC5555796

[brb370815-bib-0002] Ascherio, A. , M. G. Weisskopf , E. J. O'Reilly , et al. 2004. “Coffee Consumption, Gender, and Parkinson's Disease Mortality in the Cancer Prevention Study II Cohort: The Modifying Effects of Estrogen.” American Journal of Epidemiology 160: 977–984. 15522854 10.1093/aje/kwh312

[brb370815-bib-0003] Ascherio, A. , S. M. Zhang , M. A. Hernán , et al. 2001. “Prospective Study of Caffeine Consumption and Risk of Parkinson's Disease in Men and Women.” Annals of Neurology 50: 56–63. 11456310 10.1002/ana.1052

[brb370815-bib-0004] Badshah, H. , M. Ikram , W. Ali , S. Ahmad , J. R. Hahm , and M. O. Kim . 2019. “Caffeine May Abrogate LPS‐Induced Oxidative Stress and Neuroinflammation by Regulating Nrf2/TLR4 in Adult Mouse Brains.” Biomolecules 9: 719. 31717470 10.3390/biom9110719 PMC6921022

[brb370815-bib-0005] Bandookwala, M. , A. K. Sahu , D. Thakkar , M. Sharma , A. Khairnar , and P. Sengupta . 2019. “Edaravone‐Caffeine Combination for the Effective Management of Rotenone Induced Parkinson's Disease in Rats: An Evidence Based Affirmative From a Comparative Analysis of Behavior and Biomarker Expression.” Neuroscience Letters 711: 134438. 31422100 10.1016/j.neulet.2019.134438

[brb370815-bib-0006] Bjørklund, G. , M. Peana , M. Maes , M. Dadar , and B. Severin . 2021. “The Glutathione System in Parkinson's Disease and Its Progression.” Neuroscience and Biobehavioral Reviews 120: 470–478. 33068556 10.1016/j.neubiorev.2020.10.004

[brb370815-bib-0007] Boland, B. , W. H. Yu , O. Corti , et al. 2018. “Promoting the Clearance of Neurotoxic Proteins in Neurodegenerative Disorders of Ageing.” Nature Reviews Drug Discovery 17: 660–688. 30116051 10.1038/nrd.2018.109 PMC6456907

[brb370815-bib-0008] Bowden, J. , G. Davey Smith , and S. Burgess . 2015. “Mendelian Randomization With Invalid Instruments: Effect Estimation and Bias Detection Through Egger Regression.” International Journal of Epidemiology 44: 512–525. 26050253 10.1093/ije/dyv080 PMC4469799

[brb370815-bib-0009] Bowden, J. , G. Davey Smith , P. C. Haycock , and S. Burgess . 2016. “Consistent Estimation in Mendelian Randomization With Some Invalid Instruments Using a Weighted Median Estimator.” Genetic Epidemiology 40: 304–314. 27061298 10.1002/gepi.21965 PMC4849733

[brb370815-bib-0010] Bowden, J. , and M. V. Holmes . 2019. “Meta‐Analysis and Mendelian Randomization: A Review.” Research Synthesis Methods 10: 486–496. 30861319 10.1002/jrsm.1346 PMC6973275

[brb370815-bib-0011] Boyd, R. J. , D. Avramopoulos , L. L. Jantzie , and A. S. McCallion . 2022. “Neuroinflammation Represents a Common Theme Amongst Genetic and Environmental Risk Factors for Alzheimer and Parkinson Diseases.” Journal of Neuroinflammation 19: 223. 36076238 10.1186/s12974-022-02584-x PMC9452283

[brb370815-bib-0012] Burgess, S. , J. Bowden , T. Fall , E. Ingelsson , and S. G. Thompson . 2017. “Sensitivity Analyses for Robust Causal Inference From Mendelian Randomization Analyses With Multiple Genetic Variants.” Epidemiology 28: 30–42. 27749700 10.1097/EDE.0000000000000559 PMC5133381

[brb370815-bib-0013] Burgess, S. , and S. G. Thompson . 2017. “Interpreting Findings From Mendelian Randomization Using the MR‐Egger Method.” European Journal of Epidemiology 32: 377–389. 28527048 10.1007/s10654-017-0255-x PMC5506233

[brb370815-bib-0014] Chen, J. F. , H. K. Eltzschig , and B. B. Fredholm . 2013. “Adenosine Receptors as Drug Targets–What Are the Challenges?” Nature Reviews Drug Discovery 12: 265–286. 23535933 10.1038/nrd3955 PMC3930074

[brb370815-bib-0015] Corda, D. , P. Zizza , A. Varone , B. M. Filippi , and S. Mariggiò . 2009. “The Glycerophosphoinositols: Cellular Metabolism and Biological Functions.” Cellular and Molecular Life Sciences 66: 3449–3467. 19669618 10.1007/s00018-009-0113-4 PMC11115907

[brb370815-bib-0016] Darst, B. F. , Q. Lu , S. C. Johnson , and C. D. Engelman . 2019. “Integrated Analysis of Genomics, Longitudinal Metabolomics, and Alzheimer's Risk Factors Among 1,111 Cohort Participants.” Genetic Epidemiology 43: 657–674. 31104335 10.1002/gepi.22211 PMC6687539

[brb370815-bib-0017] Davidson, W. S. , A. Jonas , D. F. Clayton , and J. M. George . 1998. “Stabilization of Alpha‐Synuclein Secondary Structure Upon Binding to Synthetic Membranes.” Journal of Biological Chemistry 273: 9443–9449. 9545270 10.1074/jbc.273.16.9443

[brb370815-bib-0018] Dexter, D. T. , and P. Jenner . 2013. “Parkinson Disease: From Pathology to Molecular Disease Mechanisms.” Free Radical Biology and Medicine 62: 132–144. 23380027 10.1016/j.freeradbiomed.2013.01.018

[brb370815-bib-0019] Emdin, C. A. , A. V. Khera , and S. Kathiresan . 2017. “Mendelian Randomization.” JAMA 318: 1925–1926. 29164242 10.1001/jama.2017.17219

[brb370815-bib-0020] Evans, D. M. , and G. Davey Smith . 2015. “Mendelian Randomization: New Applications in the Coming Age of Hypothesis‐Free Causality.” Annual Review of Genomics and Human Genetics 16: 327–350. 10.1146/annurev-genom-090314-050016 25939054

[brb370815-bib-0021] Fabelo, N. , V. Martín , G. Santpere , et al. 2011. “Severe Alterations in Lipid Composition of Frontal Cortex Lipid Rafts From Parkinson's Disease and Incidental Parkinson's Disease.” Molecular Medicine 17: 1107–1118. 21717034 10.2119/molmed.2011.00119 PMC3188884

[brb370815-bib-0022] Fernández‐Irigoyen, J. , P. Cartas‐Cejudo , M. Iruarrizaga‐Lejarreta , and E. Santamaría . 2021. “Alteration in the Cerebrospinal Fluid Lipidome in Parkinson's Disease: A Post‐Mortem Pilot Study.” Biomedicines 9: 491. 33946950 10.3390/biomedicines9050491 PMC8146703

[brb370815-bib-0023] Freitas, A. , M. Aroso , A. Barros , et al. 2021. “Characterization of the Striatal Extracellular Matrix in a Mouse Model of Parkinson's Disease.” Antioxidants 10: 1095. 34356328 10.3390/antiox10071095 PMC8301085

[brb370815-bib-0024] Frere, S. G. , B. Chang‐Ileto , and G. Di Paolo . 2012. “Role of Phosphoinositides at the Neuronal Synapse.” Sub‐Cellular Biochemistry 59: 131–175. 22374090 10.1007/978-94-007-3015-1_5 PMC3543677

[brb370815-bib-0025] Ghosh, M. , M. Balbi , F. Hellal , M. Dichgans , U. Lindauer , and N. Plesnila . 2015. “Pericytes Are Involved in the Pathogenesis of Cerebral Autosomal Dominant Arteriopathy With Subcortical Infarcts and Leukoencephalopathy.” Annals of Neurology 78: 887–900. 26312599 10.1002/ana.24512

[brb370815-bib-0026] Greco, M. F. , C. Minelli , N. A. Sheehan , and J. R. Thompson . 2015. “Detecting Pleiotropy in Mendelian Randomisation Studies With Summary Data and a Continuous Outcome.” Statistics in Medicine 34: 2926–2940. 25950993 10.1002/sim.6522

[brb370815-bib-0027] Haqqani, M. T 1977. “Crystals in Brain and Meninges in Primary Hyperoxaluria and Oxalosis.” Journal of Clinical Pathology 30: 16–18. 838867 10.1136/jcp.30.1.16 PMC476623

[brb370815-bib-0028] Heilman, P. L. , E. W. Wang , M. M. Lewis , et al. 2020. “Tryptophan Metabolites Are Associated With Symptoms and Nigral Pathology in Parkinson's Disease.” Movement Disorders 35: 2028–2037. 32710594 10.1002/mds.28202 PMC7754343

[brb370815-bib-0029] Heller, A. , and S. S. Coffman . 2019. “Crystals in the Substantia Nigra.” ACS Chemical Neuroscience 10: 3415–3418. 31257859 10.1021/acschemneuro.9b00318

[brb370815-bib-0030] Hou, Y. , X. Dan , M. Babbar , et al. 2019. “Ageing as a Risk Factor for Neurodegenerative Disease.” Nature Reviews Neurology 15: 565–581. 31501588 10.1038/s41582-019-0244-7

[brb370815-bib-0031] Hu, G. , S. Bidel , P. Jousilahti , R. Antikainen , and J. Tuomilehto . 2007. “Coffee and Tea Consumption and the Risk of Parkinson's Disease.” Movement Disorders 22: 2242–2248. 17712848 10.1002/mds.21706

[brb370815-bib-0032] Johnson, S. C. , R. L. Koscik , E. M. Jonaitis , et al. 2018. “The Wisconsin Registry for Alzheimer's Prevention: A Review of Findings and Current Directions.” Alzheimer's & Dementia: Diagnosis, Assessment & Disease Monitoring 10: 130–142. 10.1016/j.dadm.2017.11.007 PMC5755749 29322089

[brb370815-bib-0033] Leodori, G. , M. I. De Bartolo , D. Belvisi , et al. 2021. “Salivary Caffeine in Parkinson's Disease.” Scientific Reports 11: 9823. 33972579 10.1038/s41598-021-89168-6 PMC8110998

[brb370815-bib-0034] Li, H. , F. Zeng , C. Huang , et al. 2024. “The Potential Role of Glucose Metabolism, Lipid Metabolism, and Amino Acid Metabolism in the Treatment of Parkinson's Disease.” CNS Neuroscience & Therapeutics 30: e14411. 37577934 10.1111/cns.14411 PMC10848100

[brb370815-bib-0035] Liang, S. Q. , P. H. Li , Y. Y. Hu , et al. 2023. “Myeloid‐Specific Blockade of Notch Signaling Alleviates Dopaminergic Neurodegeneration in Parkinson's Disease by Dominantly Regulating Resident Microglia Activation Through NF‐κB Signaling.” Frontiers in Immunology 14: 1193081. 37680624 10.3389/fimmu.2023.1193081 PMC10481959

[brb370815-bib-0036] Liu, M. , Q. Jiao , X. Du , M. Bi , X. Chen , and H. Jiang . 2021. “Potential Crosstalk Between Parkinson's Disease and Energy Metabolism.” Aging and Disease 12: 2003–2015. 34881082 10.14336/AD.2021.0422 PMC8612621

[brb370815-bib-0037] Lobasso, S. , P. Tanzarella , D. Vergara , M. Maffia , T. Cocco , and A. Corcelli . 2017. “Lipid Profiling of Parkin‐Mutant Human Skin Fibroblasts.” Journal of Cellular Physiology 232: 3540–3551. 28109117 10.1002/jcp.25815

[brb370815-bib-0038] Lorenz, E. C. , C. J. Michet , D. S. Milliner , and J. C. Lieske . 2013. “Update on Oxalate Crystal Disease.” Current Rheumatology Reports 15: 340. 23666469 10.1007/s11926-013-0340-4 PMC3710657

[brb370815-bib-0039] Luo, H. , W. Luo , N. Ding , et al. 2024. “Glycerophosphoinositol Modulates FGA and NOTCH3 in Exercise‐Induced Muscle Adaptation and Colon Cancer Progression.” Frontiers in Pharmacology 15: 1430400. 39130639 10.3389/fphar.2024.1430400 PMC11310102

[brb370815-bib-0040] Molina, J. A. , F. J. Jiménez‐Jiménez , P. Gomez , et al. 1997. “Decreased Cerebrospinal Fluid Levels of Neutral and Basic Amino Acids in Patients With Parkinson's Disease.” Journal of the Neurological Sciences 150: 123–127. 9268238 10.1016/s0022-510x(97)00069-5

[brb370815-bib-0041] Morelli, M. , A. R. Carta , A. Kachroo , and M. A. Schwarzschild . 2010. “Pathophysiological Roles for Purines: Adenosine, Caffeine and Urate.” Progress in Brain Research 183: 183–208. 20696321 10.1016/S0079-6123(10)83010-9 PMC3102301

[brb370815-bib-0042] Nalls, M. A. , C. Blauwendraat , C. L. Vallerga , et al. 2019. “Identification of Novel Risk Loci, Causal Insights, and Heritable Risk for Parkinson's Disease: A Meta‐Analysis of Genome‐Wide Association Studies.” Lancet Neurology 18: 1091–1102. 31701892 10.1016/S1474-4422(19)30320-5 PMC8422160

[brb370815-bib-0043] Panyard, D. J. , K. M. Kim , B. F. Darst , et al. 2021. “Cerebrospinal Fluid Metabolomics Identifies 19 Brain‐Related Phenotype Associations.” Communications Biology 4: 63. 33437055 10.1038/s42003-020-01583-z PMC7803963

[brb370815-bib-0044] Qiu, J. , G. Peng , Y. Tang , et al. 2022. “Lipid Profiles in the Cerebrospinal Fluid of Rats With 6‐Hydroxydopamine‐Induced Lesions as a Model of Parkinson's Disease.” Frontiers in Aging Neuroscience 14: 1077738. 36742201 10.3389/fnagi.2022.1077738 PMC9895836

[brb370815-bib-0045] Reichmann, H 2023. “ [Caffeine, Chocolate and Adenosine A2A Receptor Antagonists in the Treatment of Parkinson's Disease].” Fortschritte Der Neurologie‐Psychiatrie 91: 256–261. 35584767 10.1055/a-1785-3632

[brb370815-bib-0046] Reyes, R. C. , G. F. Cittolin‐Santos , J. E. Kim , et al. 2016. “Neuronal Glutathione Content and Antioxidant Capacity Can be Normalized In Situ by *N*‐Acetyl Cysteine Concentrations Attained in Human Cerebrospinal Fluid.” Neurotherapeutics 13: 217–225. 26572666 10.1007/s13311-015-0404-4 PMC4720670

[brb370815-bib-0047] Ridler, C 2018. “Parkinson Disease: Truncated α‐Synuclein Causes Mitochondrial Toxicity.” Nature Reviews Neurology 14: 252. 10.1038/nrneurol.2018.33 29545627

[brb370815-bib-0048] Ross, G. W. , R. D. Abbott , H. Petrovitch , et al. 2000. “Association of Coffee and Caffeine Intake With the Risk of Parkinson Disease.” JAMA 283: 2674–2679. 10819950 10.1001/jama.283.20.2674

[brb370815-bib-0049] Saiki, S. , Y. Sasazawa , M. Fujimaki , et al. 2019. “A Metabolic Profile of Polyamines in Parkinson Disease: A Promising Biomarker.” Annals of Neurology 86: 251–263. 31155745 10.1002/ana.25516 PMC6772170

[brb370815-bib-0050] Slob, E. A. W. , and S. Burgess . 2020. “A Comparison of Robust Mendelian Randomization Methods Using Summary Data.” Genetic Epidemiology 44: 313–329. 32249995 10.1002/gepi.22295 PMC7317850

[brb370815-bib-0051] Song, X. , M. Singh , K. E. Lee , R. Vinayagam , and S. G. Kang . 2024. “Caffeine: A Multifunctional Efficacious Molecule With Diverse Health Implications and Emerging Delivery Systems.” International Journal of Molecular Sciences 25: 12003. 39596082 10.3390/ijms252212003 PMC11593559

[brb370815-bib-0052] Storey, J. D. , and R. Tibshirani . 2003. “Statistical Significance for Genomewide Studies.” PNAS 100: 9440–9445. 12883005 10.1073/pnas.1530509100 PMC170937

[brb370815-bib-0053] Storm, C. S. , D. A. Kia , M. M. Almramhi , et al. 2021. “Finding Genetically‐Supported Drug Targets for Parkinson's Disease Using Mendelian Randomization of the Druggable Genome.” Nature Communications 12: 7342. 10.1038/s41467-021-26280-1 PMC8688480 34930919

[brb370815-bib-0054] Thamilselvan, V. , M. Menon , and S. Thamilselvan . 2009. “Oxalate‐Induced Activation of PKC‐Alpha and ‐Delta Regulates NADPH Oxidase‐Mediated Oxidative Injury in Renal Tubular Epithelial Cells.” American Journal of Physiology‐Renal Physiology 297: F1399–F1410. 19692488 10.1152/ajprenal.00051.2009 PMC2781341

[brb370815-bib-0055] Tysnes, O. B. , and A. Storstein . 2017. “Epidemiology of Parkinson's Disease.” Journal of Neural Transmission 124: 901–905. 28150045 10.1007/s00702-017-1686-y

[brb370815-bib-0056] Ulanowska, K. , J. Piosik , A. Gwizdek‐Wiśniewska , and G. We Grzyn . 2005. “Formation of Stacking Complexes Between Caffeine (1,2,3‐Trimethylxanthine) and 1‐Methyl‐4‐Phenyl‐1,2,3,6‐Tetrahydropyridine May Attenuate Biological Effects of This Neurotoxin.” Bioorganic Chemistry 33: 402–413. 16165186 10.1016/j.bioorg.2005.07.004

[brb370815-bib-0057] Venkatesan, D. , M. Iyer , A. Narayanasamy , K. Siva , and B. Vellingiri . 2020. “Kynurenine Pathway in Parkinson's Disease‐An Update.” Eneurologicalsci 21: 100270. 33134567 10.1016/j.ensci.2020.100270 PMC7585940

[brb370815-bib-0058] Von Seggern, M. , C. Szarowicz , M. Swanson , S. Cavotta , S. T. Pike , and J. T. Lamberts . 2020. “Purine Molecules in Parkinson's Disease: Analytical Techniques and Clinical Implications.” Neurochemistry International 139: 104793. 32650026 10.1016/j.neuint.2020.104793

[brb370815-bib-0059] Vrijsen, S. , M. Houdou , A. Cascalho , J. Eggermont , and P. Vangheluwe . 2023. “Polyamines in Parkinson's Disease: Balancing Between Neurotoxicity and Neuroprotection.” Annual Review of Biochemistry 92: 435–464. 10.1146/annurev-biochem-071322-021330 37018845

[brb370815-bib-0060] Wang, B. Y. , Y. Y. Ye , C. Qian , et al. 2021. “Stress Increases MHC‐I Expression in Dopaminergic Neurons and Induces Autoimmune Activation in Parkinson's Disease.” Neural Regeneration Research 16: 2521–2527. 33907043 10.4103/1673-5374.313057 PMC8374590

[brb370815-bib-0061] Xia, R. , and Z. H. Mao . 2012. “Progression of Motor Symptoms in Parkinson's Disease.” Neuroscience Bulletin 28: 39–48. 22233888 10.1007/s12264-012-1050-z PMC5560285

[brb370815-bib-0062] Zhang, T. , Y. An , Z. Shen , et al. 2024. “Serum Urate Levels and Neurodegenerative Outcomes: A Prospective Cohort Study and Mendelian Randomization Analysis of the UK Biobank.” Alzheimer's Research & Therapy 16: 106. 10.1186/s13195-024-01476-x PMC11088014 38730474

[brb370815-bib-0063] Zhao, Y. , Y. Lai , H. Konijnenberg , et al. 2024. “Association of Coffee Consumption and Prediagnostic Caffeine Metabolites With Incident Parkinson Disease in a Population‐Based Cohort.” Neurology 102: e209201. 38513162 10.1212/WNL.0000000000209201 PMC11175631

